# *Ficus dubia* latex extract prevent DMH-induced rat early colorectal carcinogenesis through the regulation of xenobiotic metabolism, inflammation, cell proliferation and apoptosis

**DOI:** 10.1038/s41598-022-19843-9

**Published:** 2022-09-14

**Authors:** Rentong Hu, Weerachai Chantana, Pornsiri Pitchakarn, Subhawat Subhawa, Bhanumas Chantarasuwan, Piya Temviriyanukul, Teera Chewonarin

**Affiliations:** 1 Department of Biochemistry, Faculty of Medicine, Chiang Mai University, 110 Intravaroros Rd., Sripoom, Muang, Chiang Mai 50200 Thailand; 2 Department of Laboratory Medicine, The Affiliated Hospital of Youjiang Medical University for Nationalities, Baise, Guangxi 533000 China; 3 Thailand Natural History Museum, National Science Museum, Klong Luang, Pathumthani 12120 Thailand; 4 Food and Nutrition Academic and Research Cluster, Institute of Nutrition, Mahidol University, Salaya, Phutthamonthon, Nakhon Pathom 73170 Thailand

**Keywords:** Biochemistry, Cancer

## Abstract

*Ficus dubia* latex is recognized as a remedy in Asian traditional medicine with various therapeutic effects. The present study aimed to determine the preventive action of *Ficus dubia* latex extract (FDLE) on 1,2-dimethylhydrazine (DMH)-induced rat colorectal carcinogenesis and its mechanisms. The experiment included an initiation model in which rats were orally administered with FDLE daily for 1 week before DMH injection until the end of the experiment, while only after DMH injection until the end in the post-initiation model. The results firstly indicated that FDLE treatment could reduce the level of methylazoxymethanol (MAM) in rat colonic lumen by inhibition of the activities of both phase I xenobiotic metabolizing enzymes in the liver and β-glucuronidase in the colon, leading to reduced DNA methylation in colonic mucosal cells, related to the number of ACF in the initiation stage. Besides, FDLE modulated the inflammation which could suppress the growth and induce apoptosis of aberrant colonic mucosal cells, leading to retardation of ACF multiplicity. Therefore, FDLE showed the ability to suppress the DMH-induced rat ACF formation and inflammation promoted growth of ACF. In conclusion, FDLE had the potential to prevent carcinogens-induced rat colorectal carcinogenesis in the initiation stage.

## Introduction

Colorectal cancer is the third most recurrently diagnosed cancer and the fourth most likely cause of cancer mortality worldwide with increasing incidence in recent years^1^. Nowadays, the chemopreventive strategies using dietary supplements and natural active compounds show potential for the protection of humans against colorectal cancer in its early stages^2,3^.

DMH-induced colorectal carcinogenesis has been established to study the chemopreventive effect of test compounds in a variety of mechanisms^4^. With regard to the DMH-induced rat model, aberrant crypt foci (ACF) are preneoplastic lesions indicated the early stages of colorectal carcinogenesis in rats and associated with colorectal cancer^5^. Whereas, inflammation is considered to be an important risk factor for the progression of many cancers^6^ and it also promotes cell proliferation by activating proliferating cell nuclear antigen (PCNA) which, as a typical marker of cell proliferation, has an important role in DNA synthesis and DNA repair during the cell cycle from G1phase to S phase^7^. Therefore, to inhibit inflammation and cell proliferation or to induce cell apoptosis becomes an effective strategy to slow down the progression of cancer^8,9^.

*Ficus dubia,* as a new *Ficus* species, is abundantly found in tropical Asia, including southern Thailand, Malaysia and Sumatra. Only a few reports have shown that its latex extract had anti-inflammatory activity in lipopolysaccharide-induced macrophages and that its sap extract had anti-oxidant activity in skin^10,11^. Especially, its latex was utilized as an ingredient in traditional drugs, due to a belief in its health benefits, unsupported by any scientific data about its anti-tumor activity. However, research has found that the anti-tumor biological activities of other *Ficus* species have been reported. For example, *Ficus racemose* contains a high content of flavonoids which had anti-diabetes, anti-inflammatory, anti-oxidant and anti-proliferation properties^12,13^. The latex of *Ficus carica* showed an inhibitory effect on the proliferation of several cancer cells^14,15^. Therefore, the present study aims to evaluate the anti-tumor efficacy of *Ficus dubia* latex extract on DMH-induced rat colorectal carcinogenesis in early stage by investigating xenobiotic metabolism, inflammation, cell proliferation and apoptosis.

## Results

### Phytochemical composition analysis of FDLE

**Figure 1:**
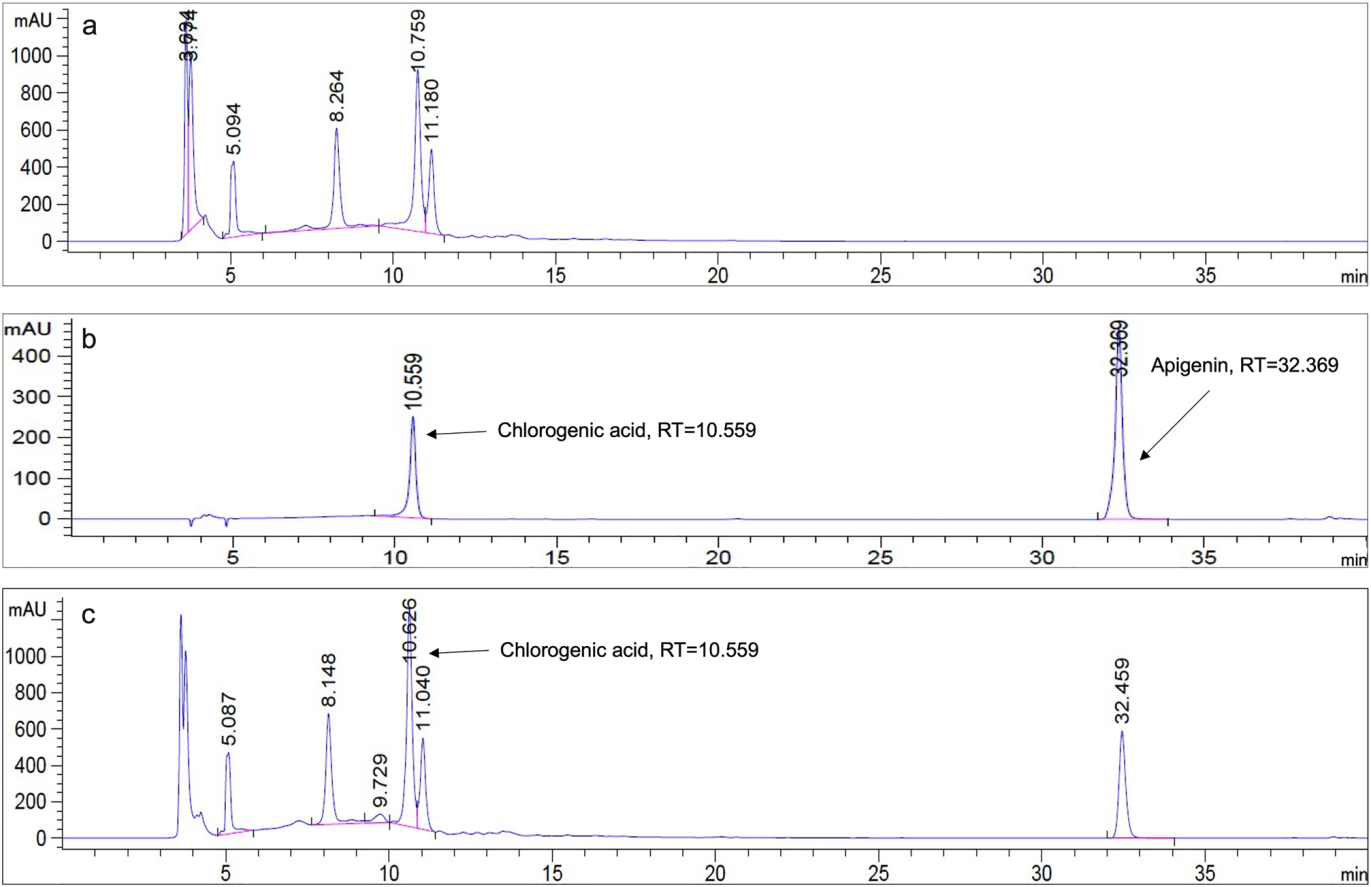
HPLC chromatograms of (**a**) FDLE, (**b**) the standard of chlorogenic acid and apigenin and (**c**) FDLE with standard.

Total amounts of phenolic acids and flavonoids in FDLE were shown in Table 1. The total phenolic and flavonoid content of FDLE were 341.6 ± 5.4 mg gallic acid equivalent (GAE)/g extract and 72.8 ± 1.5 mg catechin equivalent (CE)/g extract, respectively. The HPLC chromatogram at 280 nm (Fig. 1a–c) showed that chlorogenic acid was the predominant phenolic compound in FDLE at RT = 10.759 which was calculated to 21.1 ± 0.6 mg chlorogenic acid (CGA)/g extract while unidentified peaks were observed at RT = 5.092, 8.264 and 11.180, respectively.

**Table 1:** Total amounts of major phytochemicals in FDLE.

Phytochemicals	Total amounts (mg/g FDLE)
Total phenolic content (mg GAE)	341.6 ± 5.4
Total flavonoid content (mg CE)	72.8 ± 1.5
Chlorogenic acid (mg CGA)	21.1 ± 0.6

### Effect of FDLE on aberrant crypt foci (ACF) formation in initiation stage

**Figure 2:**
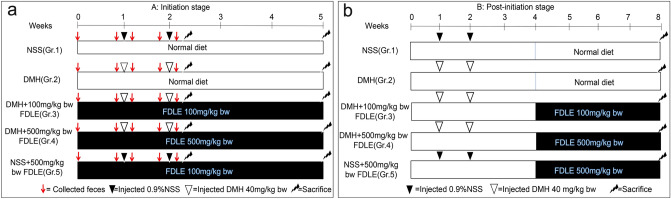
The experimental protocol and treatment schedule. (**a**) Initiation stage and (**b**) post-initiation stage of DMH-induced ACF formation.

During the entire experiment (Fig. 2a,b), the death of rats, differences of body weight and increasing level of serum ALT and AST in both initiation stage and post-initiation stage were not observed (Supplementary Table S1). The number and size of aberrant crypt foci (ACF) in experimental rats are presented in Table 2 according to the morphological structure of ACF as shown in Supplementary Fig. S1. All rats treated with DMH exhibited ACF that was distributed throughout the colon (Gr.2–4), while no ACF was observed in the NSS-treated group (Gr.1 and Gr.5). The total numbers of ACF observed in the DMH-treated group (Gr.2) accounted for 241 ± 69 (5.8% inhibition) ACF/rat. Interestingly, only administration with FDLE at dose of 500 mg/kg bw (Gr.4) significantly decreased the total number of ACF/per rat to 151 ± 41 (37.3% inhibition), when compared with a group of DMH-treated alone (Gr.2). Then, the mechanistic effect of FDLE on mutagenicity of colonic epithelial cell was investigated in the initiation stage.

**Table 2:** The number of ACF in DMH-treated rats with and without FDLE administration in initiation stage.

Treatment	Number of ACF/rat^a^				Aberrant crypt/focus
	Proximal	Distal	Rectal	Total colon (% inhibition)	
NSS (Gr.1)	0 ± 0	0 ± 0	0 ± 0	0 ± 0	0 ± 0
DMH (Gr.2)	115 ± 30	100 ± 32	26 ± 10	241 ± 69	1.6 ± 0.1
DMH + 100 mg/kg bw FDLE (Gr.3)	117 ± 29	93 ± 26	18 ± 9	227 ± 58 (5.8%)	1.5 ± 0.1
DMH + 500 mg/kg bw FDLE (Gr.4)	70 ± 24*	57 ± 15*	23 ± 10	151 ± 41* (37.3%)	1.7 ± 0.1
NSS + 500 mg/kg bw FDLE (Gr.5)	0 ± 0	0 ± 0	0 ± 0	0 ± 0	0 ± 0

^a^Mean ± SD of 6 rats per group, *p < 0.05; Significantly different from Gr.2.

### Effect of FDLE on DNA adduct and phase I and II xenobiotic metabolizing enzymes activities

**Figure 3:**
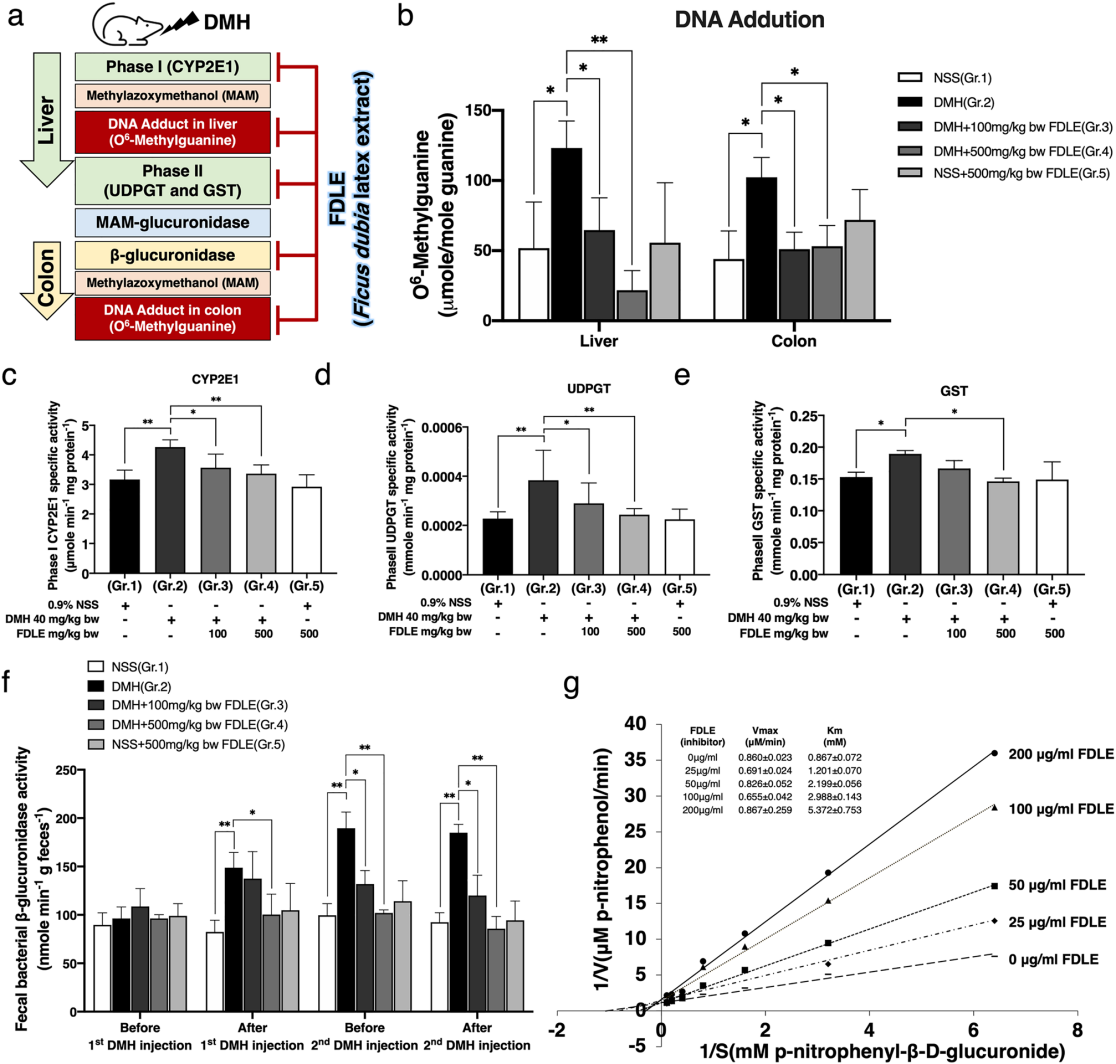
Effect of FDLE on the metabolism of dimethylhydrazine and DNA adduct formation in rat liver and colon (**a**) scheme of inhibitory effect of *Ficus dubia* latex extract on DMH-induced carcinogenesis. (**b**) The level of O^6^-MeG DNA adducts in liver tissue and colonic mucosa. (**c**) The activity of phase I and II xenobiotic metabolizing enzymes in rat liver tissue, cytochrome P4502E1 (CYP2E1) catalyzed PNP-hydroxylation, (**d**) UDP-glucuronosyltransferase (UDPGT) and (**e**) Glutathione-S-transferase (GST), phase II detoxifying enzymes. (**f**) The activity of bacterial β-glucuronidase in rat feces. (**g**) Lineweaver–Burk plot of *E. coli* β-glucuronidase in the presence of FDLE with data representing three independent experiments with similar results. Rat experiments data are presented as the mean ± SD of five rats per group. *p < 0.05, **p < 0.01.

During DMH metabolism, DNA methylation at the guanine O^6^ site (O^6^-MeG) was commonly observed in rat liver and colonic epithelial cells (Fig. 3a). The concentration of O^6^-MeG was determined 12 h after the second DMH injection in experimental rats and is presented in Fig. 3b. The O^6^-MeG level of DMH-treated rats (Gr.2) had significantly increased, when compared to the NSS-treated rats (Gr.1), in both liver tissue and colonic mucosa. In terms of FDLE-treated groups, administration at 100 and 500 mg/kg bw in DMH-treated rats (Gr.3 and 4), both significantly reduced the level of O^6^-MeG in both liver tissue and colonic mucosa, when compared to rats only treated with DMH (Gr.2), while no significant change in this number was observed in both FDLE alone and the control group.

According to a reduction of DNA methylation by MAM, the effect of FDLE on MAM generation, including activities of hepatic DMH metabolizing enzyme, CYP2E1, UDPGT and GST were determined. Figure 3c–e illustrated the activities of CYP2E1, UDPGT, and GST, respectively. When the DMH-treated group (Gr.2) was compared to the NSS-treated group (Gr.1), the activity of CYP2E1-catalized hydroxylation was dramatically increased. FDLE administration at 100 and 500 mg/kg bw in DMH-treated rats (Gr.3 and 4) all significantly reduced this activity, when compared to rats only treated with DMH (Gr.2). Additionally, the activity of UDPGT and GST were also significantly increased in DMH-injected rats (Gr.2) in the response of reactive intermediates from phase I activity. For UDPGT, FDLE administration both 100 and 500 mg/kg bw in DMH-treated rats significantly reduced the activity, when compared to rats only treated with DMH (Gr.2), whereas only FDLE at 500 mg/kg bw was able to significantly reduce GST activity. In addition, PFLE treatment alone (Gr.5) had no effect on determined xenobiotic metabolizing enzymes. These results indicated that the reduction of phase I CYP2E1 activity decreased the generation of reactive intermediate MAM and the reduction of phase II UDPGT and GST activity decreased the production of MAM conjugate, which may cause the reduction of DNA adduct levels in the liver.

### Effect of FDLE on rat fecal bacterial β-glucuronidase activity

In the colon, MAM conjugates are hydrolyzed to free MAM by intestinal bacterial β-glucuronidase enzymes, especially O^6^-methylguanine (O^6^-MeG), resulting in mutagenesis and thus tumorigenesis^16^; the activity of fecal bacterial β-glucuronidase before and after DMH injection are shown in Fig. 3f. The fecal β-glucuronidase activity in each group of rats was not different before DMH injection. After DMH injection, rat feces of the DMH-treated group (Gr.2) exhibited significantly higher β-glucuronidase activity (54.5%) compared to the NSS-treated group (Gr.1). In terms of FDLE administration, fecal β-glucuronidase activity in 500 mg/kg bw with DMH-treated group (Gr.4) significantly declined, when compared to that of the rats only treated with DMH (Gr.2). Looking at the similarities between patterns of 2nd before and after DMH injection, FDLE administration at doses of 100 and 500 mg/kg bw (Gr.3 and 4), both of which significantly decreased fecal β-glucuronidase activity compared to that of the DMH-treated alone group (Gr.2). However, only FDLE administration (Gr.5) had no effect on fecal β-glucuronidase activity, compared to the untreated group (Gr.1) in different times. The results revealed that inhibiting fecal bacterial β-glucuronidase by FDLE may result in similar effect in the liver by ultimately decreasing the level of MAM, as well as the level of DNA adducts and ACF formation in the colon. Subsequently, the effect of FDLE on the kinetics of β-glucuronidase inhibition in *E. coli* was investigated.

The kinetics of *E. coli* β-glucuronidase inhibition by FDLE were analyzed and Lineweaver–Burk plots were drawn as shown in Fig. 3g. In comparison to the untreated group, FDLE had no effect on the *E. coli* β-glucuronidase V_max_, whereas all dosages of FDLE treatments increased the K_m_ values of *E. coli* β-glucuronidase in a dose-dependent manner. The results suggested that the FDLE was a competitive inhibitor against β-glucuronidase activity. These findings demonstrated that the lowering of DMH-induced O^6^-MeG DNA adduct formation in the colon was related to the regeneration of reactive intermediate MAM by bacterial β-glucuronidase. Therefore, FDLE could alter the DNA mutations and the formation of precancerous lesions during the initiation stage of colorectal carcinogenesis.

### Effect of FDLE on aberrant crypt foci (ACF) progression in post-initiation stage

The number of ACF and crypt multiplicity (aberrant crypt/focus) in each part of the large intestine were presented in Table 3. The total number of ACF and average crypt multiplicity were observed in the DMH-treated group (Gr.2): about 354 ± 52 ACF/rat and 2.4 ± 0.2 AC/focus. Interestingly, administration with FDLE at doses of 100 and 500 mg/kg bw (Gr.3 and 4) significantly decreased the total number of ACF/rat to 239 ± 53 (32.5% inhibition) and 184 ± 63 (48.0% inhibition), when compared to rats only treated with DMH (Gr.2). Administration with FDLE at dose of 100 and 500 mg/kg bw (Gr.3 and 4) also significantly decreased the average crypt multiplicity to 2.0 ± 0.2 (16.7% inhibition) and 1.9 ± 0.1 (18.4% inhibition) when compared with DMH-treated group alone (Gr.2). Afterwards, the mechanistic effect of FDLE on inflammation, cell proliferation and apoptosis related to ACF progression were investigated in the post-initiation stage.

**Table 3:** The number of ACF and AC/F in DMH-treated rats with and without FDLE administration in post-initiation stage.

Treatment	Number of ACF/rat^a^				Aberrant crypt/focus (%inhibition)
	Proximal	Distal	Rectal	Total colon (% inhibition)	
NSS (Gr.1)	0 ± 0	0 ± 0	0 ± 0	0 ± 0	0 ± 0
DMH (Gr.2)	136 ± 26	175 ± 33	43 ± 15	354 ± 52	2.4 ± 0.2
DMH + 100 mg/kg bw FDLE (Gr.3)	97 ± 28**	125 ± 39**	17 ± 12****	239 ± 53**** (32.5%)	2.0 ± 0.2**** (16.7%)
DMH + 500 mg/kg bw FDLE (Gr.4)	73 ± 33****	96 ± 30****	15 ± 13****	184 ± 63**** (48.0%)	1.9 ± 0.1**** (18.4%)
NSS + 500 mg/kg bw FDLE (Gr.5)	0 ± 0	0 ± 0	0 ± 0	0 ± 0	0 ± 0

^a^Mean ± SD of 10 rats per group, **p < 0.01, ***p < 0.001, ****p < 0.0001, Significantly different from Gr.2.

### Effects of FDLE on inflammation in rat colonic mucosal cells

**Figure 4:**
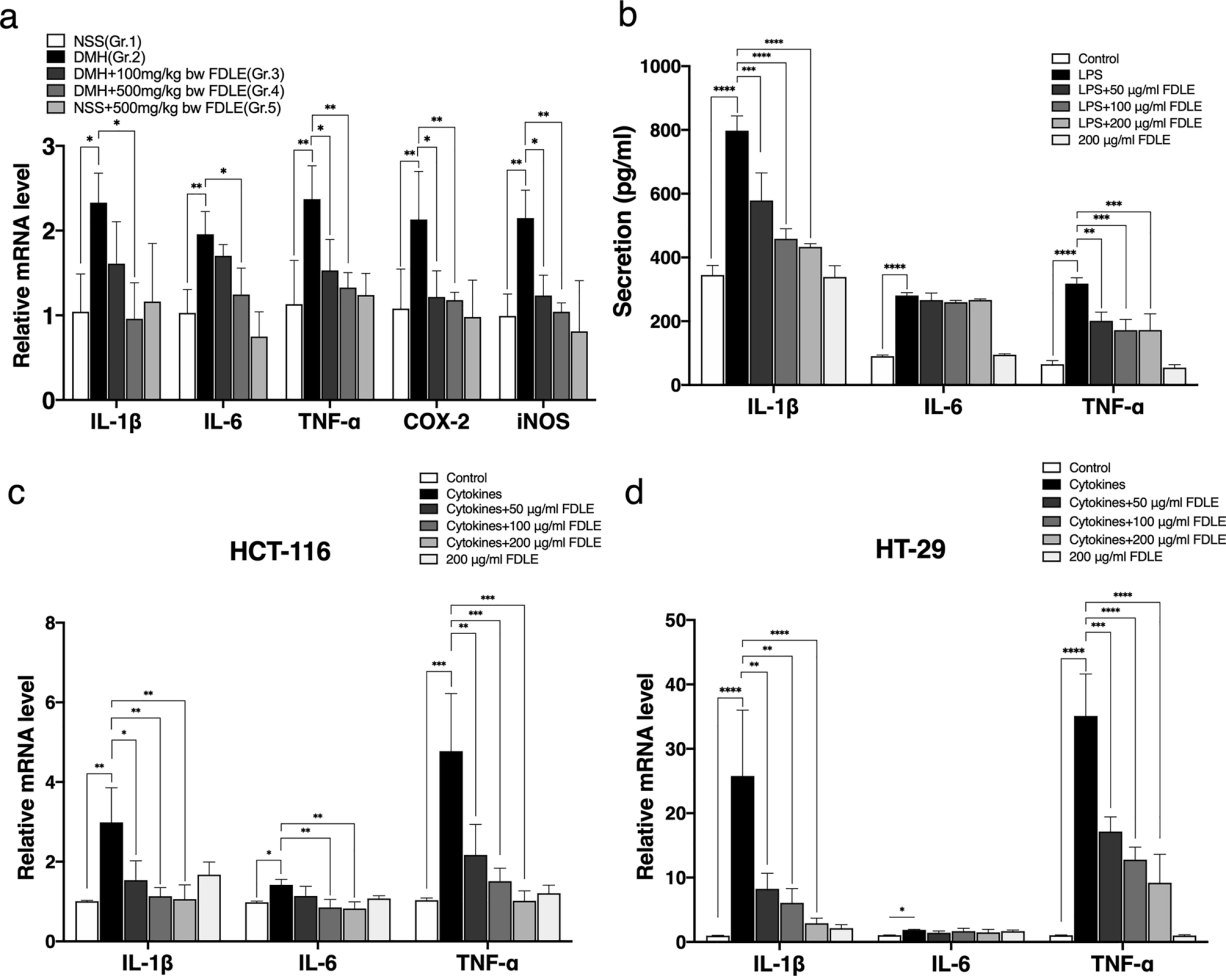
Anti-inflammation effects of FDLE (**a**) Relative mRNA level of pro-inflammatory cytokines and enzymes including IL-1β, IL-6, TNF-α, iNOS and COX-2 in rat colonic mucosa. Data are presented as the mean ± SD of 4 rats per group. (**b**) The secretion of pro-inflammatory cytokines including TNF-α, IL-1β and IL-6 in LPS induce RAW 264.7 cells treated with FDLE. Relative mRNA levels of pro-inflammatory cytokines, including TNF-α, IL-1β and IL-6 in cytokines (TNF-α, IFN-γ and LPS each 10 ng/mL) induced colorectal cancer cell lines at 48 h. (**c**) HT-29 cells. (**d**) HCT-116 cells. Data are presented as the mean ± SD of three independent experiments which are similar results. *p < 0.05, **p < 0.01, ***p < 0.001, ****p < 0.0001.

Inflammation plays a vital role in mechanism of cancer development^17^. Firstly, the mRNA levels of pro-inflammatory cytokines and enzymes in rat colonic epithelial cells were determined, as shown in Fig. 4a. The relative expression of IL-1β, IL-6, TNF-α, iNOS and COX-2 in DMH treatment (Gr.2) were significantly upregulated, compared to NSS-treated rats (Gr.1). Administration with FDLE only at a dose of 500 mg/kg bw (Gr. 4) to DMH-treated rats were able to significantly suppress the expression of IL-1β, IL-6 when, while both doses at 100 and 500 mg/kg bw of FDLE (Gr.3–4) were found to considerably suppress the expression of TNF-α, iNOS and COX-2, when compared with DMH-treated rats (Gr.2). Administration with FDLE (Gr.5) had no effect on the expression of these genes. The results suggested that FDLE could reduce the rat colonic inflammation determined by the downregulation of IL-1β, IL-6, TNF-α, COX-2 and iNOS genes expression. However, the scraped colonic mucus layer was composed of both activated immune cells and colonic epithelial cells, the inflammatory responses in the colon cancer cell line and activated macrophage were confirmedly determined in vitro.

### Effects of FDLE on inflammatory responses in cell lines

To elucidate the inhibitory effect on microenvironments rich in inflammatory cells by using LPS-activated RAW 264.7 cells, the level of pro-inflammatory cytokines TNF-α, IL-1β and IL-6 were measured, as shown in Fig. 4b. LPS increased TNF-, IL-1, and IL-6 secretion, relative to the control group, but were not compared to FDLE alone. While FDLE significantly reduced TNF- and IL-1 secretion but had no effect on IL-6 secretion compared to LPS alone. This data assumed that FDLE could reduce inflammation in macrophage induced by bacterial lipopolysaccharide.

To investigate whether FDLE could modulate the response of human colorectal cancer cell lines to mixtures of TNF-α, IFN-γ and LPS, the mRNA expression of TNF-α, IL-1β and IL-6 in HT-29 and HCT-116 in human colorectal cancer cell lines treated by a combination of cytokines was then measured and shown in Fig. 4c,d. After 48 h of treatment with a cytokine mixture, TNF-, IL-1, and IL-6 relative mRNA increased in HT-29 and HCT-116 colorectal cancer cells, compared to the negative control. FDLE reduced both TNF- and IL-1 expression in HT-29 cells, and TNF-, IL-1, and IL-6 expression in HCT-116 cells. The results indicated that FDLE also inhibited the responsibility of colorectal cancer cell lines to inflammatory cytokines from inflammation, which might be related to the growth promotion of aberrant colonic cells.

### Effects of FDLE on cell proliferation and apoptosis of rat colonic mucosa in post-initiation stage

**Figure 5:**
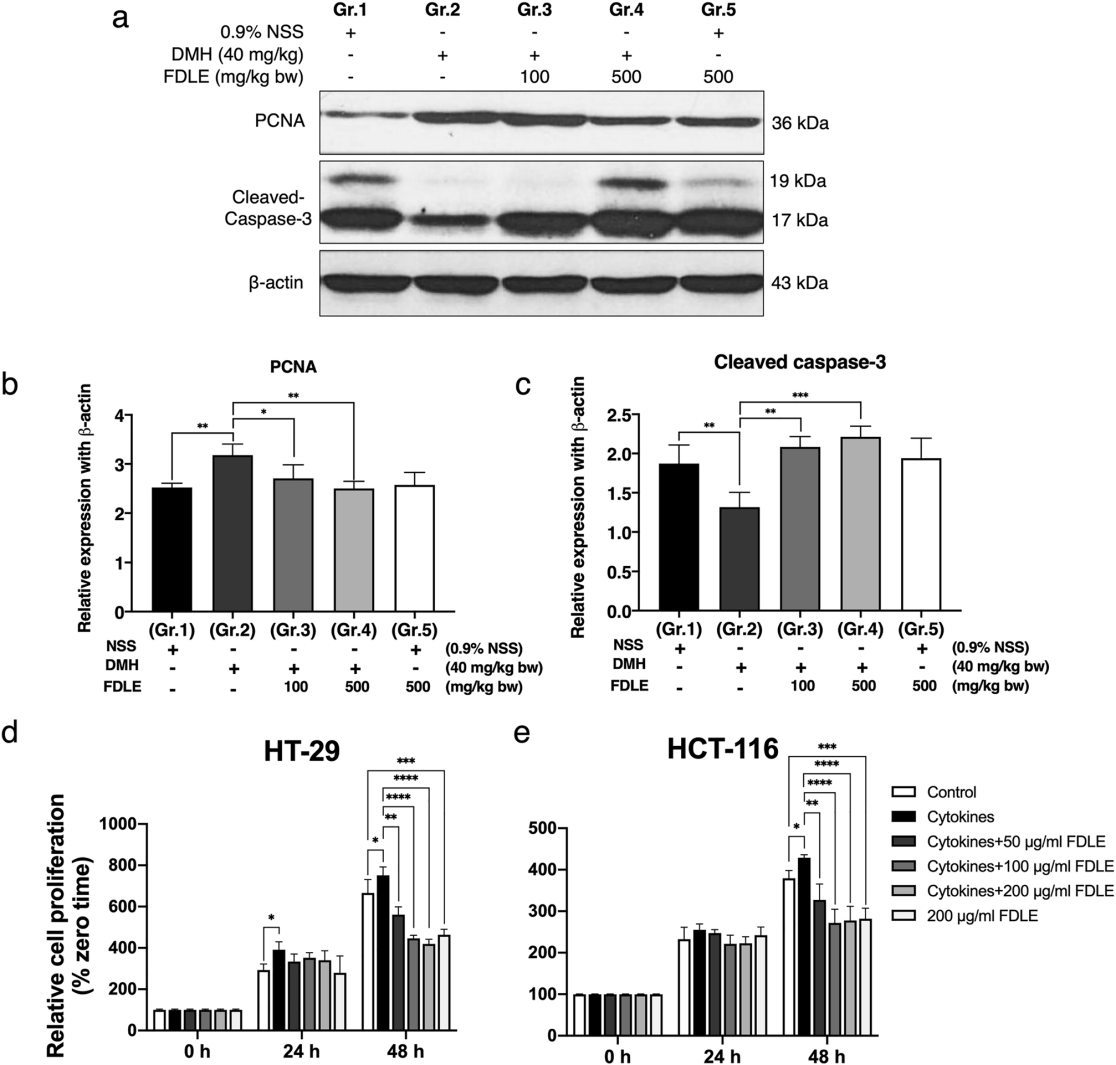
Effect of FDLE on cell proliferation and apoptosis in rat colonic mucosa and human colorectal cancer cells. (**a**) Immunoblot analysis of PCNA, cleaved caspase-3 and β-actin proteins. (**b**) Relative expression of PCNA to β-actin proteins. (**c**) Relative expression of cleaved caspase-3 to β-actin protein. Data are presented as the mean ± SD of four rats per group. Relative cell proliferation (%zerotime) of colorectal cancer cell lines with cytokines (TNF-α, IFN-γ and LPS each 10 ng/mL) or together with FDLE for 24 and 48 h. (**d**) HT-29 cells. (**e**) HCT-116 cells. Data are presented as the mean ± SD of three independent experiments with similar results, *p < 0.05, **p < 0.01, ***p < 0.001, ****p < 0.0001.

Inflammation plays a critical role in the neoplastic process by promoting proliferation and survival which inhibits apoptosis induction^18^. The protein expression related to cell proliferation and apoptosis in colonic mucosal cells was shown in the Fig. 5a–c. PCNA, cell proliferation marker protein, considerably higher in the DMH-treated group (Gr.2) compared to the NSS-treated group (Gr.1). FDLE at 100 and 500 mg/kg bw reduced the expression of PCNA protein in DMH-treated rats (Gr.3 and 4) compared to DMH alone (Gr.2). Furthermore, DMH administration retarded colonic epithelial cell apoptosis observed by reducing cleavage caspase-3 level, compared to NSS treatment (Gr.1). FDLE at 100 and 500 mg/kg bw significantly increased the level of cleaved caspase-3 protein in DMH-treated rats (Gr.3 and 4) compared to DMH alone (Gr.2). Likewise, rats only treated with FDLE (Gr.5) had no effect on the level of PCNA or cleaved caspase-3 proteins. These results confirmed that FDLE was able to inhibit cell proliferation and promote cell apoptosis in hyperproliferative colonic epithelial cells.

### Effects of FDLE on cell proliferation of HT-29 and HCT-116 colorectal cancer cell lines induced by pro-inflammatory cytokines

To investigate the effect of FDLE on the growth of the HT-29 and HCT-116 colorectal cancer cell lines in an inflammatory environment, we examined FDLE's anti-proliferation activity against cytokine-induced HT-29 and HCT-116. As shown in Fig. 5d,e, treatment with cytokines resulted in a considerable increase in the relative cell number of HT-29 and HCT-116 at 48 h, when compared to the negative control. After 48 h, FDLE administration considerably decreased cytokine-induced HT-29 and HCT-116 cell proliferation, compared to the positive control; at the same time, a high dose of FDLE administration dramatically decreased cytokine-induced HT-29 and HCT-116 cell proliferation, compared to the negative control. Cytokines were found to enhance cell proliferation, whereas FDLE inhibited cell proliferation in both inflamed and non-inflamed HT-29 and HCT-116 colorectal cancer cell lines.

## Discussion

*Ficus* species are a rich source of polyphenolic compounds and flavonoids, which are responsible for antioxidant, anti-inflammation and anti-proliferation properties that help in the prevention of various diseases^19–21^. The biological composition and function of *Ficus dubia* as a new *Ficus* species have not been studied on its which should have greater research value. Our finding showed that FDLE contains 34.2% of phenolic acids and 7.3% of flavonoids, whereas the chlorogenic acid accounted for 161.84 mg per gram of latex (2.1%) and a comparable quantity of total phenolic content and extraction yield of 7.67% from previous study^10^. Additionally, the aforementioned study provides similar results for quinic acid, a metabolite form of chlorogenic acid. The remaining phenolic acids detected in FDLE require further investigation to determine their identity. In terms of other *Ficus* species, especially *F. carica*, the most common phenolic chemicals were chlorogenic acid, followed by rutin despite the plant's extraction from other parts^22–24^. Chlorogenic acid could increase ROS generation and decrease cell viability in human colorectal cancer cells, whereas quinic acid, a chlorogenic derivative, exhibited anticancer effects in HT-29 human colorectal cancer cells in vitro and xenograft models^25,26^. Moreover, it may be a major active ingredient in *Ficus dubia* latex, which could be used to prevent liver and prostate cancer as a functional natural substance^27^. Therefore, the effect of FDLE on DMH-induced rat colorectal carcinogenesis was investigated by designed concentration according to existing of chlorogenic acid in the extract and non-toxic range (LD50 > 5000 mg/kg bodyweight).

Based on the initiation of colorectal carcinogenesis, the transformation of normal crypts to aberrant crypt foci (ACF) is a hallmark of DMH-induced colorectal carcinogenesis that has been used in a variety of studies to evaluate the extract's effects^4^. Moreover, in both animal and human, ACF are the earliest identifiable lesions and a critical morphological feature in the early stages of colorectal carcinogenesis which can be recognized on the colon mucosa^28,29^. ACF has been utilized to evaluate the preventative effects of natural compounds on the early stages of colorectal carcinogenesis^30^. Earlier evidence suggested that the bioactive compounds which inhibit ACF could also promote the anti-carcinogenicity of carcinogen-induced colorectal cancer models^31–33^. In our study, no adverse effects were found in the rats only treated with FDLE throughout an 8-week period, indicating that FDLE had not toxicity in this experimental model, as shown in Supplementary Table S1. ACF is mainly reflected in the appearance of small crypt multiplicity, which indicates the initiation of colorectal cancer. However, the progression of ACF is manifested in the transformation of small into large crypt multiplicity, which shows the progression of colorectal cancer^34^. Our results demonstrated that FDLE treatment diminished not only the total number of ACF, but also the size of crypts, particularly those with a large crypt multiplicity. Therefore, it could be firstly summarized that FDLE suppressed ACF formation at the initiation stage of DMH-induced colorectal carcinogenesis and prevented ACF progression during the post-initiation stage.

In DMH-induced rat ACF formation, liver CYP2E1 activate DMH to form methylazoxymethanol (MAM) that interacts with DNA, resulting in DNA adduction at O^6^-methylguanine (O^6^-MeG) and N^7^-methylguanine (N^7^-MeG) and then, ultimately, occur mutagenesis and tumorigenesis^16,35^. Therefore, reducing phase I enzyme activity is considered to be an effective strategy to prevent DNA adduct formation by reactive intermediates in the early stage^35–37^. It was found that DMH administration significantly increased the activities of CYP2E1 enzymes subsequently increased the level of O^6^-MeG DNA adduct in the liver, whereas the administration with FDLE significantly reduced these effects (Fig. 3b,c). UDPGT and GST detoxifying enzymes that increase the water solubility and decrease the toxicity of xenobiotic substrates by conjugation with glucuronic acid and glutathione, respectively^38^ were also determined. Unfortunately, the activities of these phase II enzymes were decreased which responded to the inhibition of phase I enzyme (Fig. 3d,e). Consequently, the data indicated that FDLE decreases DNA adducts in liver primarily by inhibiting the activity of phase I enzyme which relates to the formation of MAM.

In colon lumen, intestinal bacterial β-glucuronidase hydrolyzes the conjugated MAM to release free MAM^39^. After it is reabsorbed to colonic epithelial cell, the colorectal carcinogenesis is proceeded by DNA adduct and mutation^40^. Therefore, reducing bacterial β-glucuronidase activity is an effective strategy to prevent colorectal carcinogenesis^36,41^. In this study, administration with FDLE significantly reduced the activity of bacterial β-glucuronidase induced by DMH in the rat colon (Fig. 3f) and FDLE was confirmed to be a competitive inhibitor against purified *E.coli* β-glucuronidase (Fig. 3g). Therefore, administration with FDLE significantly reduced the colon O^6^-MeG DNA adduct by lowering free MAM from bacterial enzyme hydrolysis. All the mentioned results can be concluded that FDLE inhibited DMH-induced colorectal carcinogenesis through the alteration of xenobiotic metabolizing processes in both the liver and colon, which causes the reduction of DNA adduct levels and ACF numbers in the initiation stage. Likewise, Chlorogenic acid has been also reported to inhibit ACF formation in rat-received azoxymethane (AOM) by affecting the AKT/mTOR pathway^42,43^. Therefore, the well-known effect of chlorogenic acid has been used to compare the effects of FDLE in this study.

In post-initiation stage, DMH was administered to rats before FDLE treatment to determine its suppressive activities on ACF progression^4^. Due to the difference in ACF progression time, the amount of DMH-induced ACF in the post-initiation stage was greater than in the initiation stage, while FDLE administration still significantly decreased, accounting for 32% and 48% at doses of 100 and 500 mg/kg bw, respectively. Moreover, FDLE showed inhibitory effect against ACF multiplicity (Table 3). Therefore, in-depth investigation of the mechanism was interesting to investigate. It has been established that inflammation promotes tumor progression and pro-inflammatory cytokines such as TNF-, IL-6, and IL-1 can cooperate to regulate colorectal cancer-associated cell proliferation and differentiation, immunity, metabolism and metastatic behavior^44,45^. According to certain research, the expression of these inflammatory cytokines is increased during carcinogenesis; hence, inhibition of inflammation using certain active substances serves as an effective approach to prevent colorectal cancer^44,46,47^. Our experiment also found that DMH significantly increased the inflammatory response, while treatment with FDLE markedly decreased the expression of TNF-α, IL-6, IL-1β, iNOS and COX-2 in rat colonic mucosa, substantiating their inhibitory action towards inflammation-associated colorectal tumor progression (Fig. 4a). In addition, the anti-inflammation of FDLE on macrophages was also analyzed. FDLE significantly decreased LPS-induced secretion of TNF-α and IL-1β from macrophages (Fig. 4b). Moreover, mixed cytokines could induce inflammatory responses in HT-29 and HCT-116 colorectal cancer cell lines (Fig. 4c,d), which could promote these cancer proliferations (Fig. 5d,e). It could be demonstrated that inflammation and an inflammatory microenvironment could continue to promote cell inflammation and proliferation^48^. Interestingly, FDLE suppressed the expression of pro-inflammatory cytokines and enzymes induced by carcinogens or inflammatory inducers, which could establish their anti-inflammatory and anti-proliferative potential. Our experimental findings support the concept that inhibitors of one or some of these inducible pro-inflammatory cytokines are potential chemopreventive agents against colorectal carcinogenesis in preclinical models^49^. Correspondingly, chlorogenic acid has also been reported to decrease the inflammatory response by decreasing TNF-α and COX-2 levels^50^.

The balance of cell proliferation and apoptosis is critical for the intestinal mucosa's integrity, and dysregulation of tumor cell proliferation and apoptosis frequently results in hyperplasia and carcinogenesis^2,46,51^. Therefore, inhibiting cell proliferation and inducing cell apoptosis are considered to be effective strategies for the prevention and treatment of cancer^9^. In this experiment DMH dramatically raised nuclear availability of PCNA protein, a marker of DNA synthesis, leading to increase cellular proliferation of colonic epithelial cells which related to aberration of colonic crypt alignment in DMH-treated rat according to the morphological structure of ACF (Supplementary Fig. S1). Administration with FDLE significantly decreased the expression of PCNA protein (Fig. 5a,b) with the amelioration of ACF (Supplementary Fig. S1). In addition, colorectal cancer cell lines proliferation was response to combinations of cytokines whereas this growth was suppressed by FDLE treatments (Fig. 5d,e). In terms of apoptosis, DMH dramatically reduced the activation of caspase-3, but an increase of cleaved caspase 3 was observed following FDLE administration, demonstrating that FDLE may enhance cell apoptosis (Fig. 5a,d). Moreover, these effects are most likely the result of chlorogenic acid that suppresses cell proliferation and induces apoptosis^43,52^. When combined with the results of previous inflammation and ACF in vivo and in vitro experiments, these data clearly demonstrated that FDLE inhibited the multiplicity of ACF by controlling inflammation, which leads to decreased cell proliferation and induces cell apoptosis in the post-initiation stage and this plant will be value-added further in the future.

## Conclusion

**Figure 6:**
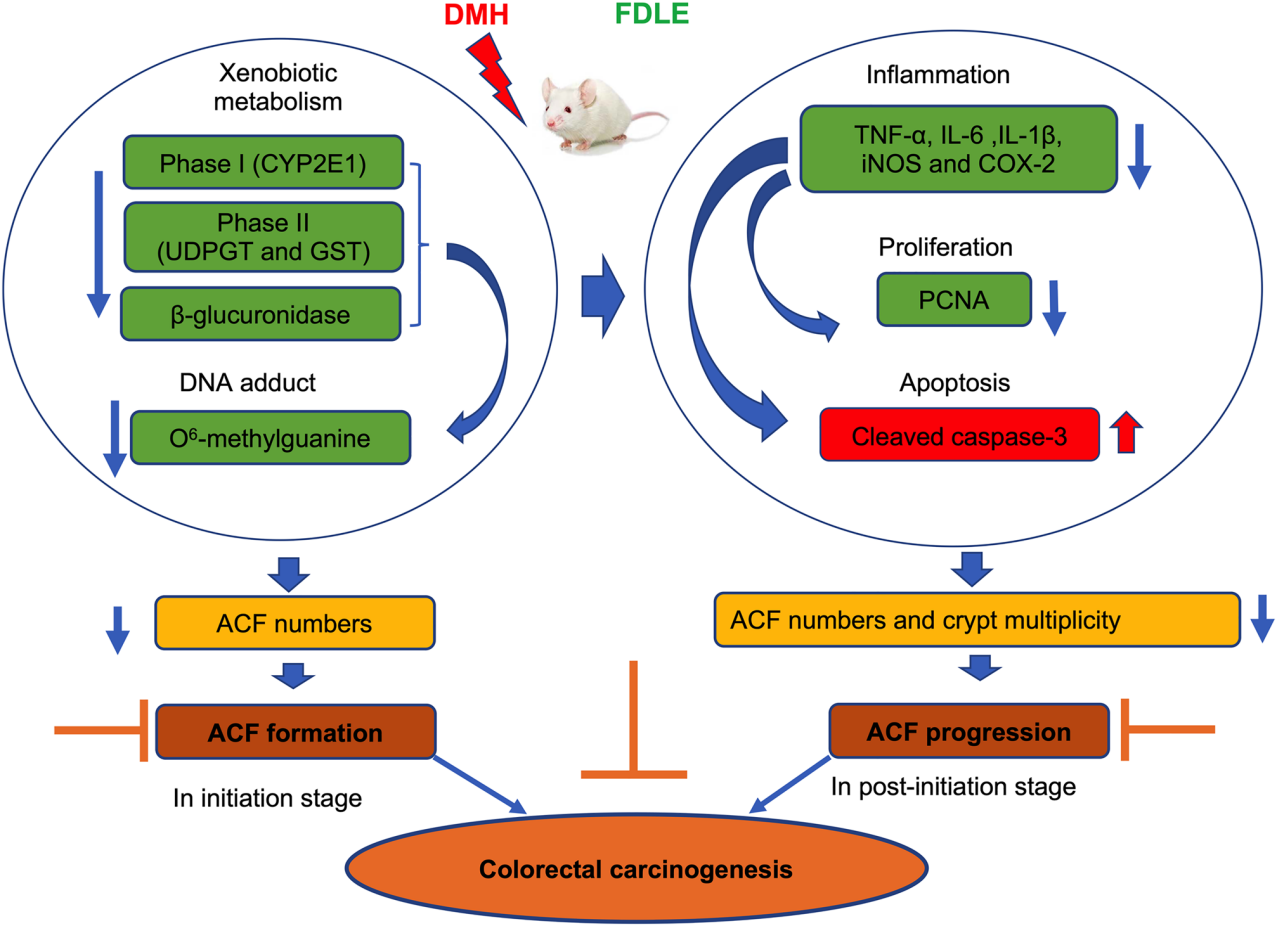
The chemopreventive mechanism of FDLE in DMH-induced rat colorectal carcinogenesis in the early stage.

The experimental findings of our study indicate that administration of FDLE effectively prevent DMH-induced rat colorectal carcinogenesis in the early stage by the inhibition of ACF formation and progression. FDLE inhibits ACF formation by reducing xenobiotic metabolizing enzymes activities in the liver and reducing β-glucuronidase activity in the colon, leading to both reduced formation of DNA adducts and the number of ACF, in the initiation stage. FDLE inhibits ACF progression by reducing inflammation, leading to decreased cell proliferation and increased cell apoptosis which caused a decrease of ACF numbers and crypt multiplicity in the post-initiation stage. Therefore, the latex of *Ficus dubia* has the potential to be a chemopreventive agent for colorectal diseases. The molecular mechanisms and the applications should be further investigated. A summary of chemopreventive mechanism of FDLE in DMH-induced rat colorectal carcinogenesis in the early stage is shown in Fig. 6.

## Materials and methods

All methods in these studies were performed in accordance with the relevant guidelines and regulations. In addition, the animal protocol has been proved by the Faculty of Medicine, Chiang Mai University, Thailand (code: 04/2562). We confirmed that all data were reported in accordance with ARRIVE guidelines.

### Chemicals

The carcinogen 1, 2-dimethylhydrazine (DMH) was obtained from Tokyo Chemical Industry. P-nitrophenyl-β-d-glucuronide, *E coli* β-glucuronidase, glutathione, uridine diphosphate glucuronic acid (UDPGA), 1-chloro-2,4dinitro benzene (CDNB), glycogen, guanine and O^6^-MeG were purchased from Sigma-Aldrich Chemical Co. Tris–HCl and sucrose were obtained from Thermo Fisher Scientific Inc. Phenol–chloroform–isoamylalcohol was obtained from Research Organics Inc. Other reagent grade chemicals were obtained from Merck Millipore Bioscience (Thailand).

### The extraction and preparation of *Ficus dubia* latex

*Ficus dubia* latex was collected and identified by Bhanumas Chantarasuwan, National Science Museum, Thailand (voucher number: Chantarasuwan 040117-1) and extract was conducted and provided by the Institute of Nutrition, Mahidol University as described in previous study^10^. The powder of *Ficus dubia* latex was soaked with deionized water at the ratio of 1:10 w/v. The aqueous fraction was filtrated and freeze-dried. The crude extract powder of Ficus dubia latex (FDLE) was kept at − 20 °C for further experiments. FDLE was dissolved in deionized water just before use.

### Measurement of total phenolic and flavonoid contents

The total phenolic contents were determined by Folin–Ciocaiteu assay, as described in Song et al.^53^. The total phenolic contents were calculated and expressed as gallic acid equivalents (GAE) in mg/g extract, by comparison with the standard calibration curve of gallic acid. The total flavonoid contents were determined by aluminum chloride colorimetric method. The assay was performed according to the method of Subedi et al.^54^ The total flavonoid contents were calculated and expressed as catechin equivalents (CE) in mg/g extract by comparison with the standard calibration curve of catechin.

### Measurement of known phenolic and flavonoid concentration

The phenolic and flavonoid fingerprint of FDLE were determined by HPLC, according to the method of Shao et al.^55^. The extract was determined for the existence of phytochemical contents by HPLC using a C18 column (250 × 4.6 mm, 5 μm) (Agilent Technologies, Santa Clara, CA, USA). The chromatographic separation was carried out using the isocratic mode of mobile phase A (1% acetic acid in water) and mobile phase B (100% acetonitrile) with a total run time of 50 min for detection with a flow rate of 0.7 mL/min. The gradient system used was 90% A in 0 min–60% in 28 min, followed by 40% in the next 39 min and 10% in the next 50 min. The extract of 10 mg/mL dissolved in 1 mL of MeOH was injected into the column and phytochemical contents at 280 nm were detected. The peak area and retention time of the extract sample were evaluated as the comparison with the standard curve (apigenin and chlorogenic acid).

### Animals model and protocol approving

The male Wistar rats (4–5 weeks of age, weighing 80–100 g) used in this study were purchased from Nomura Siam International, Thailand. Rats were housed in cages in an air-conditioned room (25–30 °C) with exposure to 12/12-h light/dark cycle. The animal treatment protocol was approved by the Animal Ethics Committee, Faculty of Medicine, Chiang Mai University, Thailand (code: 04/2562).

### Colorectal cancer induction and experimental protocol and treatment schedule

#### Initiation protocol

Rats were divided into five groups (10 rats each). The control groups (groups 1 and 5) received a normal diet (negative control) and only a high dose of FDLE (treatment control) with 0.9% normal saline solution (NSS) injection, respectively. In the experimental groups, positive control (group 2) rats received a normal diet. Rats in group 3 and 4 received FDLE of 100 and 500 mg/kg bodyweight (equal to chlorogenic acid 2.1 and 10.5 mg/kg body weight), respectively, for 1 week before the first injection of DMH and continued for 5 weeks. All experimental rats were injected with DMH (40 mg/kg of body weight), subcutaneously, once a week at weeks 1 and 2. Feces of rats from each group were directly collected for β-glucuronidase activity measurement. Twelve hours after the second DMH injection (2 weeks), five rats of each group were sacrificed using Zoletil 100, a combination of tiletamine and zolazepam, at 20 mg/kg of body weight. The liver and colon were collected for xenobiotic metabolism enzyme activity and DNA adduct determination. After 5 weeks, the remaining six rats were sacrificed, then the colon was collected and processed for ACF determination according to Bird et al.^28^. The whole colon was collected and defined as having three parts including proximal colon (caecum, ascending colon, transverse colon), distal colon (descending colon and sigmoid colon) and rectum(rectal) according the description by Keum^56^ as shown in Supplementary Fig. S2. Serum was used for measurement of ALT and AST for liver function.

#### Post-initiation protocol

The grouping was the same as the initiation stage with 14 rats/group, FDLE were treated after 2 weeks of the second DMH injection for a further 4 weeks. After the rats were sacrificed, colons from six rats were fixed for determination of ACF and those from the other four rats were frozen for mRNA and protein extraction. The toxicity of FDLE was evaluated by rat body weight and serum transaminase activity, sGOT and sGPT.

### Aberrant crypt foci (ACF) analysis

Rat colons were removed and fixed in 10% formalin-phosphate-buffer saline (PBS) solution (pH 7.4) by initial cutting the rectum (2 cm from the anus) followed by the proximal and distal convoluted tubule halving of the colon. Fixed colons were stained with 0.2% blue methylene, then the ACF were scored under a light microscope at a magnification of 10 ×. The number of ACF/rat and AC/focus were analyzed according to Bird et al.^28^.

### Measurement of phase I and phase II xenobiotic metabolic enzymes activity

Liver tissues were extracted cytosolic and microsomal protein by centrifugal sedimentation^33^. The procedure for microsomal cytochrome P4502E1 (CYP2E1)-catalyzed p-nitrophenol hydroxylation measurement was based on the method described by Chang et al., with some modifications^57^, the enzyme activity was calculated and expressed as micromole of product formed/min/mg protein. UDP-Glucuronosyltransferase (UDPGT) activity measurement was done using p-nitrophenyl phosphate (PNP) as the substrate according to the modified method of Woodcock et al.^58^, while glutathione-S-transferase (GST) activity measurement was evaluated by monitoring the conjugation of 1-chloro-2,4-dinitro benzene (CDNB) with glutathione, according to the methods of Habig et al.^59^.

### Measurement of fecal bacterial β-glucuronidase activity

Fecal bacterial β-glucuronidase activity was measured using rat feces collected before and after the injections of DMH, using p-nitrophenyl phosphate (PNP) as the substrate, according to the modified method of Deeptha et al*.*^60^. The enzyme activity was calculated and expressed as μmol min^−1^/g feces. To determine the kinetically inhibitory effect of FDLE *E. coli* β-glucuronidase, 5 µL of 20 U/mL of *E. coli* β-glucuronidase was added to a reaction mixture containing 5 µL of 0.1 M PBS pH 7.0, 5 µL of 0.1 mM EDTA with the 5 µL of various concentration of FDLE and incubated at 37 °C for 15 min. The reaction was started by adding 5 µL of 2–8 mM of *p*-nitrophenyl-β-d-glucuronide (PNPG) substrate or distilled water (blank), then the reaction mixture was incubated at 37 °C for 15 min. After adding 200 µL of 0.2 M glycine buffer, pH 10.4 in 0.2 M NaCl, absorbance was measured at 450 nm to calculate the reaction velocity in the presence of each concentration of inhibitor (extract). The alteration of K_m_ and V_max_ were determined from double-reciprocal plot to define the inhibition type of FDLE against β-glucuronidase.

### Measurement of DNA adducts O^6^-methylguanine by HPLC

The procedure for detection of DNA adducts was based on the method described by Iwitzki et al.^61^ Frozen liver and colon were defrosted on ice. Total DNA was isolated by phenol–chloroform and DNA in the aqueous phase was precipitated with cold ethanol. 500 μg of DNA was hydrolyzed in 1 M HCl at 95 °C for 1 h (acid hydrolysis). Diluted DNA hydrolysate was analyzed using an Agilent HPLC Column (PL-SCX1000A 10 μm 250 × 4.6 mm). The isocratic separation with buffer A (10% methanol in 20 mM ammonium formate, pH 4.0) was carried out for 3 min then a linear gradient was applied ranging from buffer A to 25% buffer B (5% methanol in 200 mM ammonium formate, pH 4.0) for 15 min, followed by 25% buffer B for 2 min at a flow rate of 1.5 mL/min. The nucleotide bases were detected using a UV detector at 275 nm. The amount of hydrolyzed DNA was quantified by the peak area of the guanine and O^6^-methylguanine. The level of O^6^-methylguanine was calculated by using a standard curve and expressed as micromole of O^6^-MeG/mole of guanine.

### Determination of pro-inflammatory cytokines and enzymes mRNA expression by qPCR

Total RNA from rat colonic mucosa was isolated using Trizol reagent (AMBION, USA) following the manufacturer’s instructions. The cDNA was synthesized from total RNA (2 µg) using ReverTra Ace qPCR RT master mix (TOYOBO, Japan) in a 20 µL reaction mixture carried out at 37 °C for 15 min, 50 °C for 5 min, 98 °C for 5 min. PCR amplification performed with the maxima SYBR Green qPCR master mix (Thermo Fisher Scientific, USA), the reaction included at 95 °C for 15 min, 95 °C for 15 s, 60 °C for 60 s for 40 cycles and the PCR-amplified gene products were analyzed. The specific primer parings were; TNF-α fwd 5′-AAATGGGCTCCCTCTCATCAGTCC-3′, rev 5′-TCTGCTTGGTGGTTTGCTACGAC-3′; IL-6 fwd 5′-TCCTACCCCAACTTCAATGCTC-3′, rev 5′-TTGGATGGTCTTGGTCCTTA-GCC-3′; IL-1β: fwd 5′-CACCTCTCAAGCAGAGCACAG-3′, rev 5′-GGGTTCCATGGT-GAAGTCAAC-3′; COX-2 fwd 5′-GCCCACCAACTTACAATGTGC-3′, rev 5′-CATGGG-AGTTGGGCAGTCAT-3′ and iNOS fwd 5′-CATTGGAAGTGAAGCGTTTCG-3′, rev 5′-CAGCTGGGCTGTACAAACCTT-3′ were quantified with normalization to β-actin mRNA fwd 5′-GACATGCCGCCTGGAGAAAC-3′, rev 5′-AGCCCAGGATGCCCTTTAGT-3′. Level of mRNAs were quantified with normalization to β-actin mRNA.

### Measurement of pro-inflammatory cytokine in culture supernatant by ELISA

Firstly, RAW264.7 cells (5 × 10^5^ cells) were cultured in a 6-well plate with 1 µg/mL lipopolysaccharide (LPS) for 24 h. Then, the cells were washed with PBS and various concentrations of PFLE (50, 100 and 200 µg/mL) were added for 24 h. Finally, the culture supernatant was collected and used to determine the secreted inflammatory cytokines including TNF-α, IL-1β and IL-6 with ELISA kit (Thermo Fisher Scientific, USA and Canada) according to the manufacturer’s instruction. Three independent experiments were performed with at least triplicate per experiment.

### Measurement of cell proliferation by MTT

HT-29 and HCT-116 colon cancer cells (1 × 10^4^ cells) were cultured in a 96-well plate with cytokines (TNF-α, IFN-γ and LPS at each 10 ng/mL) for 24 h, then, various concentrations of FDLE (50, 100 and 200 µg/mL) were added for 24 and 48 h. At the indicated times, 15 µL of 5 mg/mL MTT solution were added. The incubation was carried out at 37 °C for 4 h. All solution was discarded and a 100 µL DMSO was added to dissolve formazan crystals. The violet solution was measured the absorbance at 540/630 nm by using microplate reader. A percentage of cell proliferation was calculated against vehicle treated cells. The experiments were repeated three times with at least triplicate per experiment.

### Measurement of PCNA and cleaved caspase-3 by western blot

Total proteins were collected after centrifugation of epithelial cell homogenate and SDS-polyacrylamide gel electrophoresis (PAGE) was used to separate denatured proteins and then transferred them onto nitrocellulose membrane. The transferred proteins were detected using specific primary anti-PCNA (1:2000), anti-cleaved caspase-3 (1:1000), anti-β-actin (1:5000) overnight at 4 °C and secondary anti-rabbit (1:5000) were labeled with horse radish peroxidase enzyme followed by chemiluminescent substrate. The specific target proteins were visualized by adding Western Lightening Chemiluminescent HRP Substrate (PerkinElmer, USA) and the pictures were captured by Kodak X-ray film. The intensity of the target proteins was measured by ImageJ program. The relative expression was normalized against β-actin expression and then compared to the non-treatment group.

### Statistical analysis

The values are given as the mean ± SD. Significant differences between the means of the groups were analyzed by one way analysis of variance (ANOVA). The results were considered statistically significant at p < 0.05, *p < 0.05, **p < 0.01, ***p < 0.001, ****p < 0.0001. All the image quantifications were done by Image-Pro (version 7.0). All the statistics were performed on GraphPad Prism 6.0 (GraphPad Software, USA).

## Supplementary Information

Supplementary Figures.

Supplementary Tables.
