# Supplementary material for: Ficus dubia latex extract prevent DMH-induced rat early colorectal carcinogenesis through the regulation of xenobiotic metabolism, inflammation, cell proliferation and apoptosis
Source: Sci Rep. 2022 Sep 14;12:15472. doi: 10.1038/s41598-022-19843-9 (PMC9474822; doi:10.1038/s41598-022-19843-9)

**Supplementary Figure**

**Figure S1** Morphology of ACF in rat colon after stained with 0.2% methylene blue


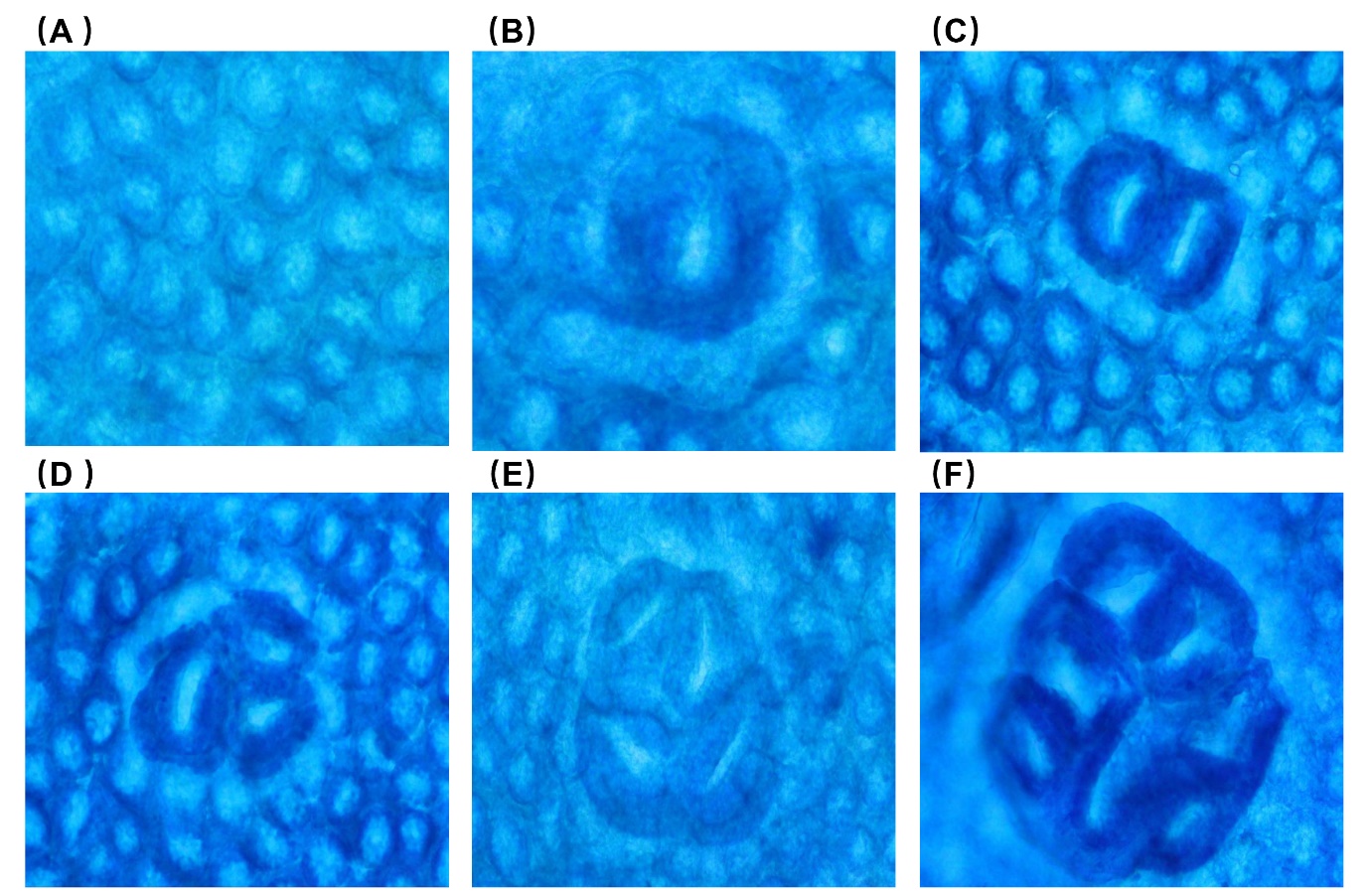


1. Normal, (B) 1 crypt/focus, (C) 2 crypt/focus, (D) 3 crypt/focus, (E) 4 crypt/focus,

(F) multiple crypt/focus

The photo was taken under microscope with 40X magnification.

**Figure S2** Whole of the colon and colon section preparation


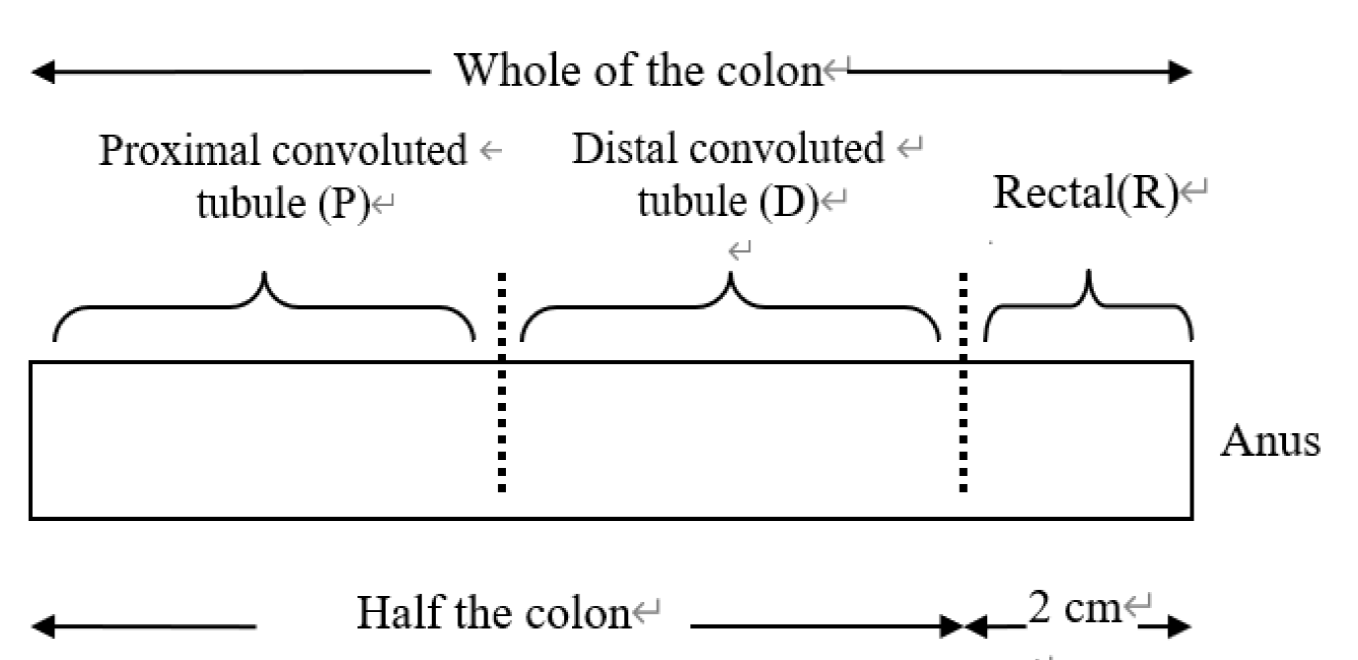

Supplement: Supplementary file 1 — Supplementary Figures. [file 41598_2022_19843_MOESM1_ESM.docx]
