# Supplementary material for: Ficus dubia latex extract prevent DMH-induced rat early colorectal carcinogenesis through the regulation of xenobiotic metabolism, inflammation, cell proliferation and apoptosis
Source: Sci Rep. 2022 Sep 14;12:15472. doi: 10.1038/s41598-022-19843-9 (PMC9474822; doi:10.1038/s41598-022-19843-9)
Supplement: Supplementary file 2 — Supplementary Tables. [file 41598_2022_19843_MOESM2_ESM.docx]

**Supplementary Table**

***Ficus dubia* latex extract prevent DMH-induced rat early colorectal carcinogenesis through the regulation of xenobiotic metabolism, inflammation, cell proliferation and apoptosis**

**Authors**

Rentong Hu^1,2^, Weerachai Chantana^1^, Pornsiri Pitchakarn^1^, Subhawat Subhawa^1^, Bhanumas Chantarasuwan^3^, Piya Temviriyanukul^4^, Teera Chewonarin^1,*^

**Affiliations**

^1^ Department of Biochemistry, Faculty of Medicine, Chiang Mai University, 110 Intravaroros Rd., Sripoom, Muang, Chiang Mai 50200, Thailand

^2^ Department of Laboratory Medicine, The Affiliated Hospital of Youjiang Medical University for Nationalities, Baise 533000, Guangxi, China

^3^ National Science Museum, Technopolish, Klong 5, Klong Luang, Pathumthani, Thailand

^4^ Food and Nutrition Academic and Research Cluster, Institute of Nutrition, Mahidol University, Salaya, Phuttamonthon, Nakhon Pathom, Thailand

**Table S1** The changes of rat death, body weight, serum ALT and AST in initiation stage and in post-initiation stage

| Treatment | In initiation stage | | | | | In post-initiation stage | | | |
| --- | --- | --- | --- | --- | --- | --- | --- | --- | --- |
|  | Death | Body weight(g) | ALT (U/L) | AST (U/L) | Death | | Body weight(g) | ALT (U/L) | AST (U/L) |
| NSS (Gr.1) | 0 | 343±18 | 29±3 | 94±8 | 0 | | 484±18 | 35±7 | 57±5 |
| DMH (Gr.2) | 0 | 333±19 | 36±3 | 112±8 | 0 | | 472±15 | 37±5 | 79±4 |
| DMH+100mg/kg bw FDLE (Gr.3) | 0 | 324±16 | 36±3 | 106±14 | 0 | | 466±14 | 36±10 | 64±9 |
| DMH+500mg/kg bw FDLE (Gr.4) | 0 | 336±22 | 37±6 | 95±9 | 0 | | 476±14 | 33±5 | 54±3 |
| NSS+500mg/kg bw FDLE (Gr.5) | 0 | 354±22 | 37±9 | 102±25 | 0 | | 459±16 | 36±10 | 63±9 |
